# Quantitative water T2 relaxometry in the early detection of neuromuscular diseases: a retrospective biopsy-controlled analysis

**DOI:** 10.1007/s00330-022-08862-9

**Published:** 2022-05-21

**Authors:** Noah Locher, Benedikt Wagner, Fabian Balsiger, Olivier Scheidegger

**Affiliations:** 1 Centre for Neuromuscular Diseases, Department of Neurology, Inselspital, Bern University Hospital, University of Bern, Bern, Switzerland; 2 Support Center for Advanced Neuroimaging (SCAN), Institute for Diagnostic and Interventional Neuroradiology, Inselspital, Bern University Hospital, University of Bern, Bern, Switzerland; 3 Universitätsklinik für Neurologie, Inselspital, Freiburgstrasse, CH-3010 Bern, Switzerland

**Keywords:** Neuromuscular diseases, Magnetic resonance imaging, Muscles, Biopsy, Water T2 relaxation time, fat-suppressed T2-weighted MRI

## Abstract

**Objectives:**

To assess quantitative water T2 relaxometry for the early detection of neuromuscular diseases (NMDs) in comparison to standard qualitative MR imaging in a clinical setting.

**Methods:**

This retrospective study included 83 patients with suspected NMD who underwent multiparametric MRI at 3 T with a subsequent muscle biopsy between 2015 and 2019. Qualitative T1-weighted and T2-TIRM images were graded by two neuroradiologists to be either pathological or normal. Mean and median water T2 relaxation times (water T2) were obtained from manually drawn volumes of interests in biopsied muscle from multi-echo sequence. Histopathologic pattern of corresponding muscle biopsies was used as a reference.

**Results:**

In 34 patients, the T1-weighted images showed clear pathological alternations indicating late-stage fatty infiltration in NMDs. In the remaining 49 patients without late-stage changes, T2-TIRM grading achieved a sensitivity of 56.4%, and mean and median water T2 a sensitivity of 87.2% and 97.4% to detect early-stage NMDs. Receiver operating characteristic (ROC) analysis revealed an area under the curve (AUC) of 0.682, 0.715, and 0.803 for T2-TIRM, mean water T2, and median water T2, respectively. Median water T2 ranged between 36 and 42 ms depending on histopathologic pattern.

**Conclusions:**

Quantitative water T2 relaxometry had a significantly higher sensitivity in detecting muscle abnormalities than subjective grading of T2-TIRM, prior to late-stage fatty infiltration signal alternations in T1-weighted images. Normal-appearing T2-TIRM does not rule out early-stage NMDs. Our findings suggest considering water T2 relaxometry complementary to T2-TIRM for early detection of NMDs in clinical diagnostic routine.

**Key Points:**

• *Quantitative water T2 relaxometry is more sensitive than subjective assessment of fat-suppressed T2-weighted images for the early detection of neuromuscular diseases, prior to late-stage fatty infiltration signal alternations in T1-weighted images*.

• *Normal-appearing muscles in fat-suppressed T2-weighted images do not rule out early-stage neuromuscular diseases*.

• *Quantitative water T2 relaxometry should be considered complementary to subjectively rated fat-suppressed T2-weighted images in clinical practice*.

## Introduction

Magnetic resonance imaging (MRI) is widely used clinically to diagnose and monitor neuromuscular diseases (NMDs). Late-stage disease manifestation of NMDs such as fatty infiltration can be identified in T1-weighted images by hyperintense signal alterations. Similarly, elevated T2 signals in muscle, an unspecific surrogate marker of the strength of underlying pathophysiological processes, but also of early pathological tissue alterations [1], can be identified in fat-suppressed T2-weighted images. Both alterations can be qualitatively and semi-quantitatively graded, for instance, by using the Mercuri and Fischer scales [2, 3]. However, such a qualitative and semi-quantitative grading is inherently subjective [4]. As sensitivity thresholds are unknown, it is difficult to compare between patients and interventions [5]. To overcome these limitations, quantitative MR parameters to assess pathological changes in NMDs have been introduced.

Quantitative MRI provides objective and sensitive measures of nuclear MR tissue microstructure. Quantitative MR parameters such as the percentage of fatty infiltration of muscle (fat fraction) and the muscle water T2 relaxation time (water T2) are well-established MR parameters in NMDs [5–7]. The percentage of fatty infiltration quantifies chronic degenerative changes of muscle, or more generally disease severity. Water T2 is an unspecific marker for disease activity and assesses, among others, the presence of leaky membranes, muscle fiber necrosis, edema, inflammation, or denervation. Therefore, water T2 is prolonged by early changes in muscle integrity before fatty infiltration occurs in late disease stages [1]. This might render water T2 useful for the early detection of NMDs, when subtle changes in the muscle might not appear on standard, qualitatively assessed MRI.

The early detection of disease might be beneficial for clinical care of patients with NMDs. By early detection, a potential treatment could be initiated before irreversible muscle damage has occurred. Additionally, the diagnostic yield of muscle biopsy could be increased by targeting more suitable muscles for histopathological analysis. Diagnostically, an imaging-based early detection of NMDs might either be possible by using fat-suppressed T2-weighted sequences, or by quantitative water T2 relaxometry [5, 6, 8, 9]. Although quantitative water T2 relaxometry has been studied as a potential biomarker for various NMDs [10–18], to the best of our knowledge, studies that determined the value of water T2 for early detection of NMDs in comparison to standard, qualitatively assessed, fat-suppressed T2-weighted sequences with histopathology as a reference do not exist.

We conducted a retrospective study to determine the value of quantitative water T2 relaxometry for the early detection of NMDs. We hypothesized that more early-stage NMDs could be identified by quantitative water T2 relaxometry compared to standard, qualitatively assessed, fat-suppressed T2-weighted sequences.

## Materials and methods

### Study population

Our study compromised a cohort of *n* = 83 patients (38 women, mean age 60, range 19–91) referred to our tertiary center for neuromuscular diseases between January 2015 and May 2019 due to suspected NMD (muscle weakness, atrophy, myalgia) who underwent both multiparametric muscle MRI and subsequent muscle biopsy. All patients had previously given oral and written informed consent and the study was approved by the local ethics committee.

### MR protocol

All MR examinations were performed on a 3 T Siemens MAGNETOM Skyra^fit^ (Siemens Healthineers) using 12-channel body coils and spine coils. Images of calves and thighs were acquired using the following protocol: axial T1-weighted sequences, axial T2-weighted sequences with turbo inversion recovery magnitude for fat saturation (T2-TIRM), and quantitative water T2 relaxometry derived from axial multi-slice multi-echo (MSME) sequences. T1-weighted sequences were acquired using a repetition time (TR) of 270 ms, echo time (TE) of 11 ms, field of view (FoV) read of 400 mm, FoV phase of 50%, base resolution of 384, and slice thickness of 5 mm. T2-TIRM sequences were acquired using a TR of 2020 ms, TE of 44 ms, flip angle (FA) of 140°, inversion time (TI) of 200 ms, FoV read of 400 mm, FoV phase of 50%, base resolution of 384, and slice thickness of 5 mm. Water T2 relaxometry was derived from MSME sequences with a TR of 3000 ms, 17 echoes with TEs of 9.5 ms, 19.5 ms, …, 161.5 ms, FA of 180°, FoV read of 448 mm, FoV phase of 50%, base resolution of 320, parallel imaging mode GRAPPA with factor 2, slice thickness of 5 mm, and 9 slices. The choice of the MSME sequence parameters corresponds to what is typically used in clinical neuromuscular MRI [19]. Total scan time of the protocol was 15 min 26 s.

### Muscle histopathology

All patients underwent a biopsy of skeletal muscle tissue originating from one muscle selected based on abnormalities on standard T1-weighted image, T2-TIRM image, and water T2 relaxometry map. Our clinical protocol defines that the chosen muscle should show mild abnormalities in T1-weighted and T2-TIRM images. Only if no abnormalities are present in weighted images, the water T2 relaxometry map is consulted to select a muscle with elevated water T2 values for the biopsy. Histopathological analysis of the muscle biopsies was performed according to standard protocols [20]. The biopsies were categorized by a neuropathologist into five primary histopathologic patterns:

1. neurogenic (atrophic fibers, group typing, esterase positive myocytes)
2. myopathic (increased fiber size variability, endomysial fibrosis, high amount of central nuclei, fiber splitting)
3. inflammatory (infiltration of inflammatory cells, expression of MHC-I)
4. unspecific (mild abnormalities, no clear fitting to above mentioned pattern)
5. normal (no abnormalities)

### Image analysis

The image analysis involved a subjective grading of the standard qualitative MR images, and a calculation of the mean and median of the water T2 values obtained from T2 relaxometry map. In T1-weighted and T2-TIRM images, muscles were graded to be either pathological (i.e., hyperintense signal noticeable) or normal by two radiologists. Both raters have over 5 years of experience in musculoskeletal and neuromuscular diagnostics and were blinded to the clinical data and each other’s result. In case of disagreement, the two raters consulted to reach a consensus. For water T2 relaxometry, the exponential decay of the signal was fitted voxel-wise from the MSME images using a bi-component extended phase graph (EPG)–derived signal model [19] to obtain water T2 relaxometry maps. The fitting was performed offline with Python-based software provided by the authors of [19], which is available upon reasonable request. Mean and median of the water T2 values were calculated using volumes of interests (VOIs). VOIs were manually drawn in the muscles on the MSME image with the echo time of 161.5 ms using the open-source software ITK-SNAP version 3.6 [21]. The VOIs were drawn on all nine image slices by one rater, with instructions to only include muscular tissue and to avoid vessels. Similar to the subjective grading of the T1-weighted and T2-TIRM images, the rater was blinded to the clinical data. For a comparison to the grading of the T1-weighted and T2-TIRM images, muscles were categorized as pathological or normal using a cut-off value of ≥ 35 ms (mean +2 standard deviation of water T2 values of healthy volunteers as reported in [19]). Therefore, for each patient, four gradings were derived: two subjective gradings based on muscle appearance on the T1-weighted and T2-TIRM images and two gradings based on the mean and median water T2 values of a VOI within the muscle.

### Statistical analysis

To test the hypothesis that more early-stage NMDs can be identified with quantitative water T2 relaxometry compared to T2-TIRM, patients with a pathological grading of the T1-weighted image were excluded from the statistical analysis as visible alternations in T1-weighted images indicate late-stage fatty infiltration in NMDs. In patients with normal grading of the T1-weighted images, the diagnostic performance of the subjective T2-TIRM grading and the quantitative mean and median water T2 values of the VOIs with the histopathology as a reference was assessed using sensitivity, specificity, positive predictive value (PPV), and negative predictive value (NPV). Receiver operating characteristic (ROC) curve analysis was performed and the area under the curve (AUC) was calculated for further comparison between the different gradings. To determine if there is a difference of water T2 values between the histopathologic patterns, we used the non-parametric Kruskal-Wallis and Wilcoxon rank-sum tests (small sample size and non-normal distributed data). All statistics were conducted with the level of significance at *p* < 0.05. We used R version 4.1.2 (R Core Team) to conduct all statistical analyses.

## Results

**Fig. 1:**
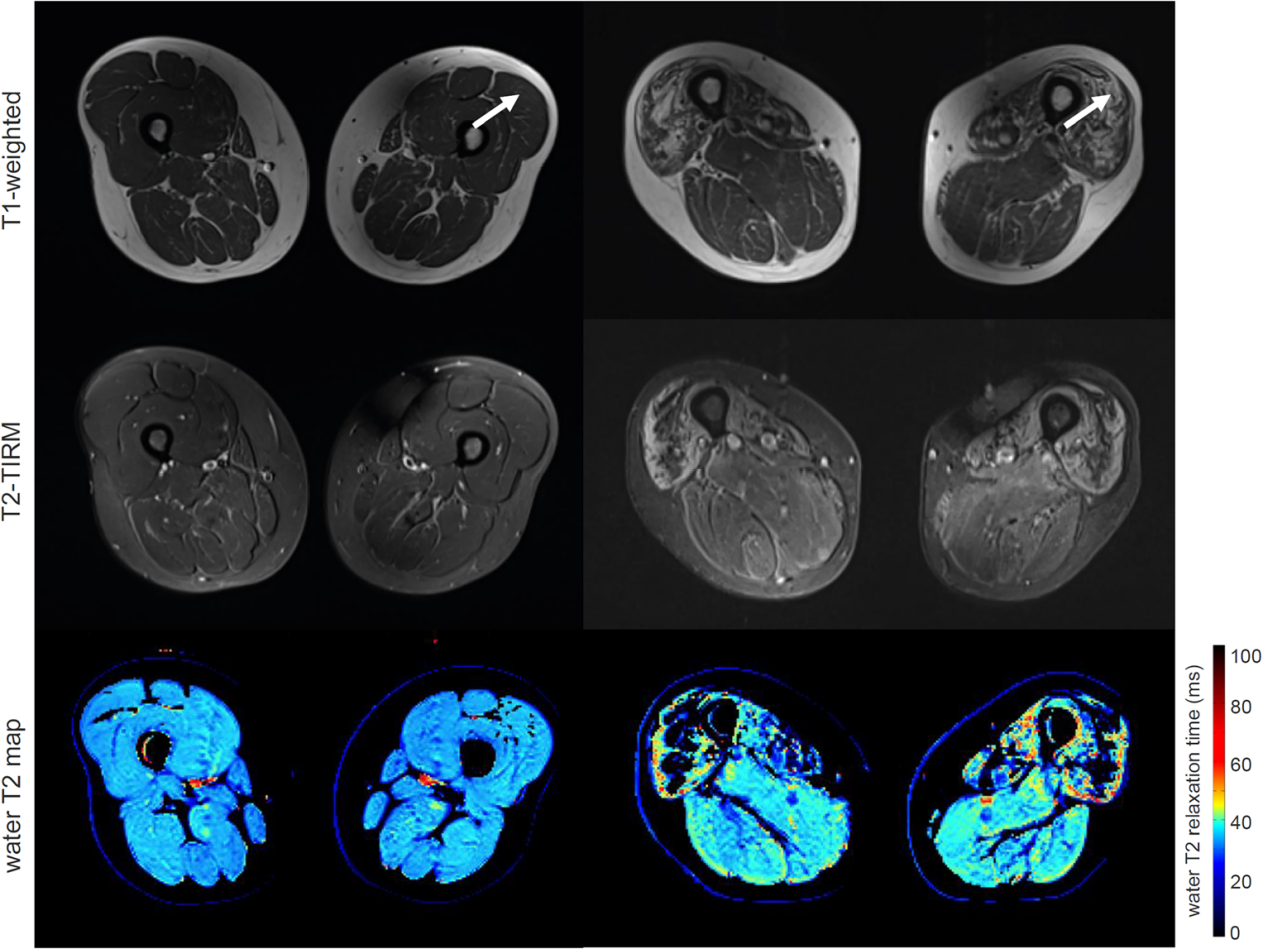
Axial sections of standard T1-weighted and T2-TIRM images, and water T2 relaxometry map (top to bottom) of two patients. Water T2 relaxation times are color-coded in water T2 maps, with cold colors depicting normal values (< 35 ms) and warm colors pathological values, respectively. (*Left*) A patient with myalgia and normal histopathological pattern (female, 44 years) showing no signal alternations in T1-weighted and T2-TIRM images and healthy water T2 values in all thigh muscles. (*Right*) A patient with inclusion body myositis (male, 64 years) showing atrophy and clear hyperintense signal alternations in T1-weighted images indicating late-stage fatty infiltration in the quadriceps muscles. T2-TIRM also shows abnormal high signal intensity, and prolonged water T2 values are also measured. Arrows indicate the muscle used for the analysis (biopsy, subjective grading, and VOI)

**Fig. 2:**
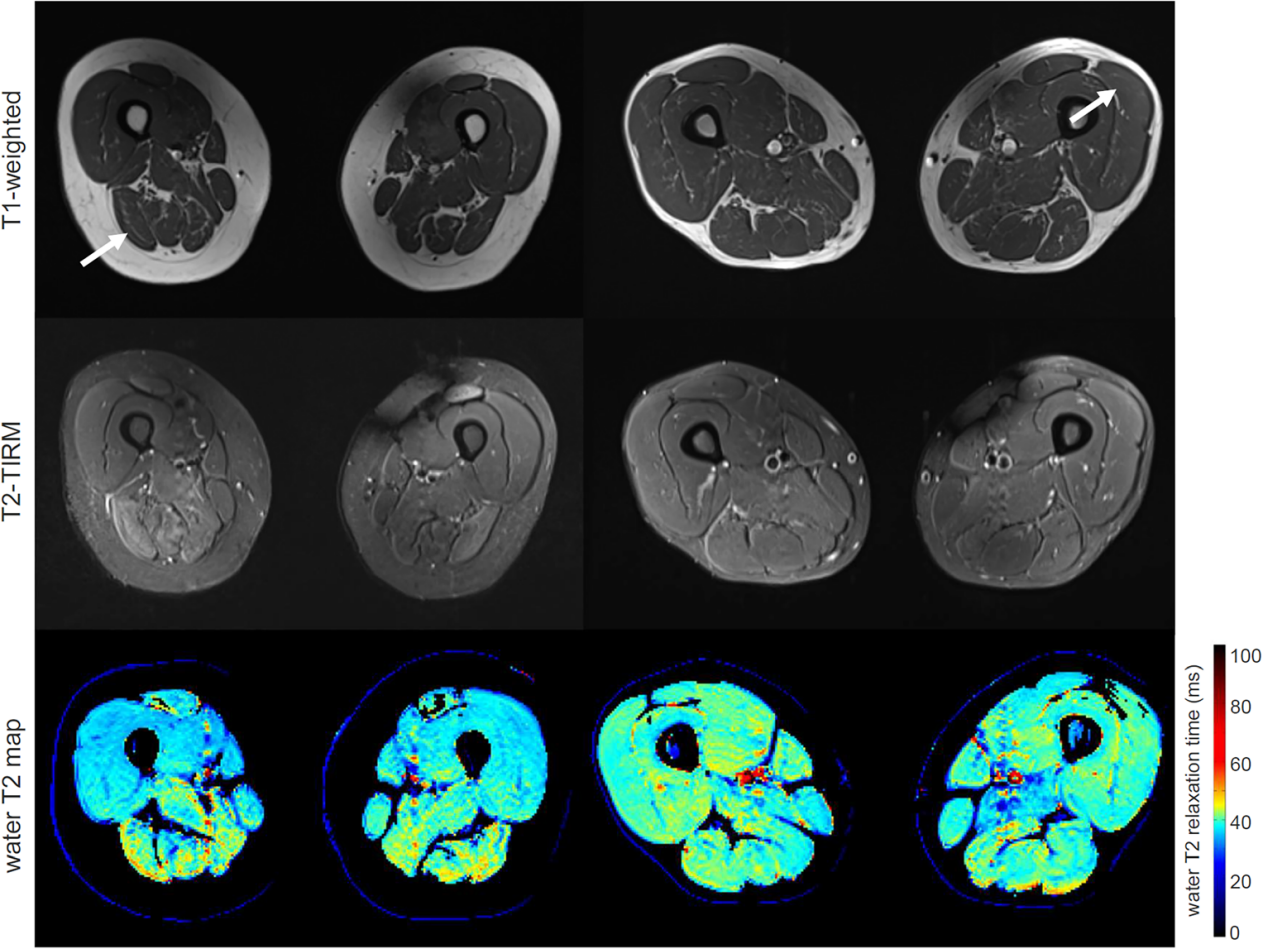
Axial sections of standard T1-weighted and T2-TIRM images, and water T2 relaxometry map (top to bottom) of two patients showing an abnormal histopathological pattern on muscle biopsy. Water T2 relaxation times are color-coded in water T2 maps, with cold colors depicting normal values (< 35 ms) and warm colors pathological values, respectively. In both patients, T1-weighted images appear normal indicating no late-stage fatty degeneration of muscle tissue. (*Left*) A patient with statin-induced necrotizing myopathy (female, 86 years) showing hyperintense signal alternations in T2-TIRM and prolonged water T2 values in the posterior compartment of the thighs. (*Right*) A patient with necrotizing autoimmune myopathy (male, 54 years) showing no clear signal alternations in T1-weighted and T2-TIRM images, but prolonged water T2 values in all thigh muscles. Normal T2-TIRM signals do not rule out disease activity indicating early stages of an NMD, as confirmed by histopathology. Arrows indicate the muscle used for the analysis (biopsy, subjective grading, and VOI)

Histopathology confirmed 70 patients to have pathological muscles in our cohort of 83 patients. In 13 patients, no pathological alterations were found. Muscle biopsies were taken from vastus lateralis (*n* = 40), biceps femoris long head (*n* = 26), adductor magnus (*n* = 2), adductor longus (*n* = 1), semimembranosus (*n* = 6), gracilis (*n* = 1), tibialis anterior (*n* = 3), and gastrocnemius (*n* = 4). In our cohort of 83 patients, 34 patients showed clear late-stage fatty infiltration based on subjective grading of the T1-weighted images, which were excluded in subsequent statistical analysis. T1-weighted images, T2-TIRM images, and water T2 relaxometry maps of a patient with normal histology and MR imaging and a patient with late-stage fatty infiltration are shown in Fig. 1. In the remaining 49 patients without late-stage changes, the distribution of the histopathological pattern was neurogenic (*n* = 15), inflammatory (*n* = 6), myopathic (*n* = 14), unspecific (*n* = 4), and normal (*n* = 10). Exemplary T1-weighted images, T2-TIRM images, and water T2 relaxometry maps of two patients without late-stage changes are shown in Fig. 2. The inter-rater agreement of the subjective grading of the standard imaging was 85.5% for T1-weighted and 88.0% for T2-TIRM images for the entire cohort.

The performances of the grading methods to detect early NMD with histopathology as a reference are summarized in Table 1. T2-TIRM grading achieved a sensitivity of 56.4%, while mean and median water T2 relaxometry achieved sensitivities of 87.2% and 97.4%. On the contrary, the specificity of T2-TIRM was higher with 80.0% compared to 40.0% and 30.0% for mean and median water T2 relaxometry.

**Table 1:** Diagnostic performances of subjective T2-weighted image grading versus quantitative T2 relaxometry. Sensitivity, specificity, positive predictive value (PPV), and negative predictive value (NPV) for the subjective grading of fat-suppressed T2-weighted turbo inversion recovery magnitude (T2-TIRM) sequence, mean water T2, and median water T2. Values in the parenthesis denote the 95% CI

Grading	Sensitivity (%)	Specificity (%)	PPV (%)	NPV (%)
T2-TIRM	56.4 (39.6–72.2)	77.8 (44.4–97.5)	91.7 (73.0–99.0)	32.0 (14.9–53.5)
Mean water T2	87.2 (72.6–95.7)	44.4 (12.2–73.8)	85.0 (70.2–94.3)	44.4 (13.7–78.8)
Median water T2	97.4 (86.5–99.9)	33.3 (6.67–65.2)	84.4 (70.5–93.5)	75.0 (19.4–99.4)

**Fig. 3:**
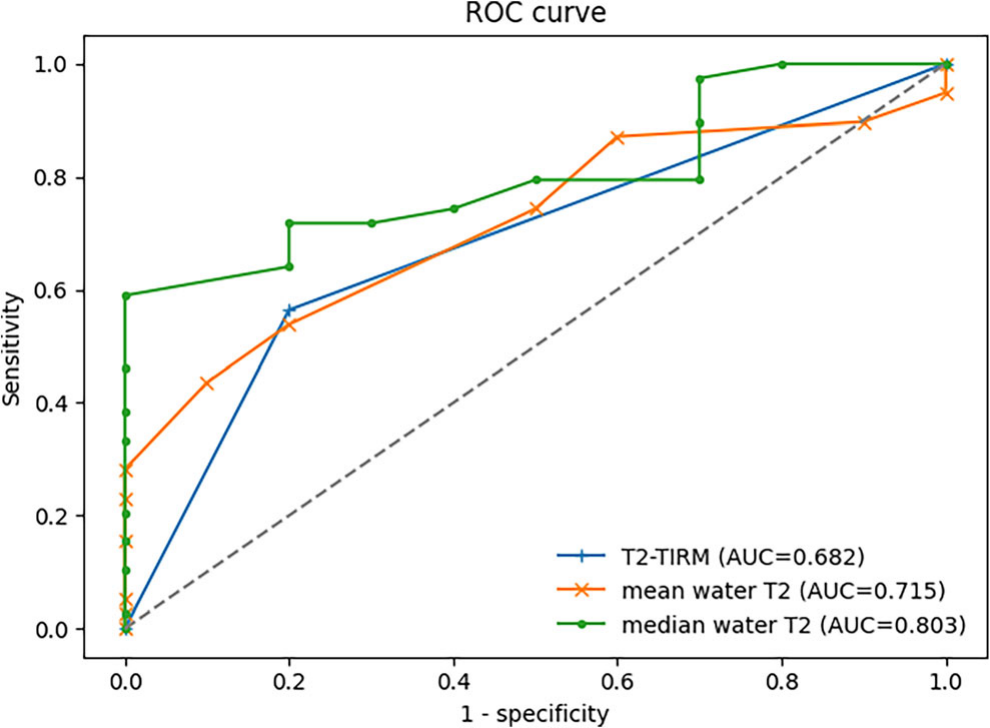
Receiver operating characteristic (ROC) curves for T2-TIRM, mean water T2, and median water T2 grading. AUC, area under the curve

The ROC analysis of the grading to discriminate pathological and normal muscle revealed an AUC of 0.682 (95% CI: 0.529–0.835) for T2-TIRM, 0.715 (95% CI: 0.560–0.871) for mean water T2, and 0.803 (95% CI: 0.674–0.931) for median water T2, as shown in Fig. 3. The AUCs indicate only a fair diagnostic ability for T2-TIRM and mean water T2 grading due to a lack of sensitivity for the T2-TIRM grading and a lack of specificity for the mean water T2 grading. However, the median water T2 grading shows a good diagnostic ability to discriminate pathological and normal muscle in early-stage NMDs.

**Fig. 4:**
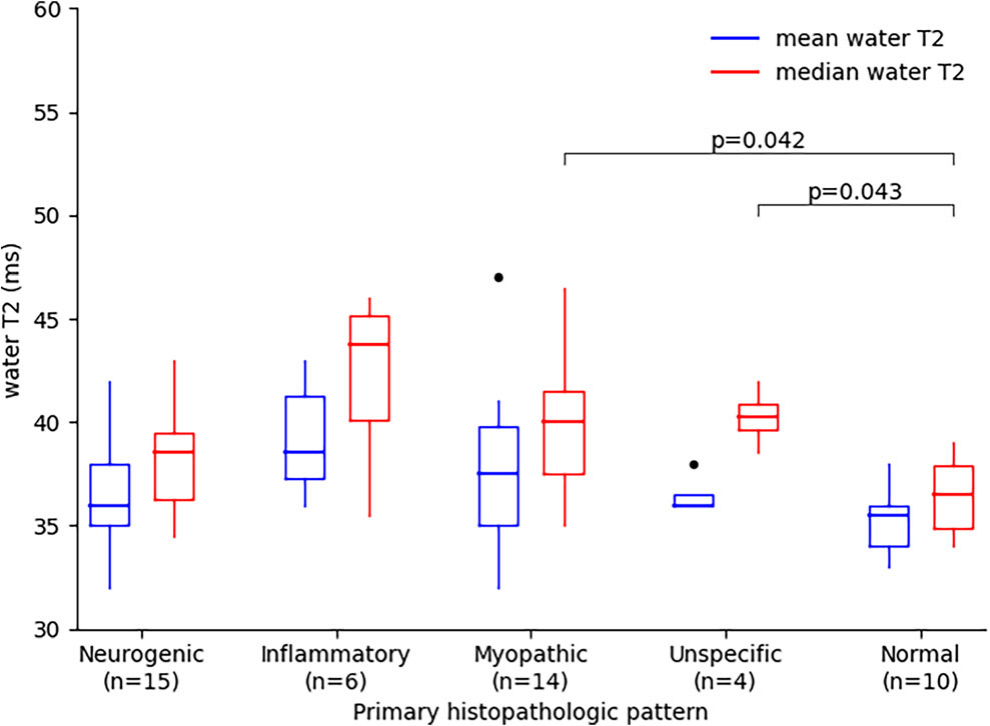
Box-and-whisker plots of the mean and median water T2 in the VOIs of the patients without late-stage changes (*n* = 49) grouped by the histopathologic patterns: neurogenic, inflammatory, myopathic, unspecific, and normal. Only median water T2 of myopathic and unspecific patterns differ statistically from normal patterns (Wilcoxon rank-sum test with Bonferroni adjustment for multiple comparisons)

Water T2 values of the histopathological pattern are shown as box-and-whisker plots in Fig. 4. For the mean water T2 values, there is no significant difference between the histopathologic patterns (*p* = 0.102). For the median water T2 values, there is a significant difference between the histopathologic patterns (*p* = 0.008). The Wilcoxon rank-sum test with Bonferroni adjustment for multiple comparisons determined that myopathic (*p* = 0.042) and unspecific (*p* = 0.043) patterns differ statistically from normal patterns but neurogenic (*p* = 0.110) and inflammatory (*p* = 0.066) patterns do not differ statistically from normal patterns. The mean and standard deviation of the mean and median water T2 were 36.7 ± 2.7 ms and 38.3 ± 2.5 ms for neurogenic, 39.2 ± 2.5 ms and 42.3 ± 3.8 ms for inflammatory, 37.5 ± 3.7 ms and 39.8 ± 3.1 ms for myopathic, 36.5 ± 0.9 ms and 40.3 ± 1.3 ms for unspecific, and 35.3 ± 1.5 ms and 36.5 ± 1.8 ms for normal pattern. The differences in mean and median water T2 are associated with an asymmetric distribution of water T2 in the VOIs of the muscles resulting in a more robust median water T2 measure. Normal patterns were found to have high water T2 mostly due to confounding vascular pathologies of the lower limbs in six patients (extensive varicosis *n* = 1, peripheral artery occlusive disease *n* = 1, congestive heart disease with edema *n* = 1, and metabolic-toxic reasons *n* = 3).

## Discussion

We determined the value of quantitative water T2 relaxometry for the early detection of NMDs compared to subjective grading of T2-TIRM images with histopathology as a reference. The median water T2 had a significantly higher sensitivity in detecting early-stage muscle abnormalities than the subjective grading of T2-TIRM images, prior to visible signal alternations in T1-weighted images. Therefore, normal-appearing muscles in T2-TIRM images do not rule out disease manifestation in muscles (cf. Fig. 2). The added value was especially noticed in cases of either borderline elevation of water T2, or homogenous elevation of water T2 in all muscles of an anatomical region leading to no discernible image contrast between individual muscles in T2-TIRM images [8]. The higher sensitivity of water T2 could help in selecting optimal sites for muscle biopsy and may therefore lead to more conclusive histopathological findings, faster diagnosis, and therapy commencement if available. Therefore, quantitative water T2 relaxometry has a role in the diagnostic workup of NMDs in detecting early abnormalities beyond its current use as an outcome measure [5, 7].

Water T2 relaxometry in muscles has been used as a biomarker in natural history studies of neuromuscular diseases but requires further considerations for clinical application [5, 6]. First, elevated water T2 is an unspecific finding related to different pathophysiological neuromuscular processes such as leaky membranes, muscle fiber necrosis, inflammation, or denervation [1]. Hence, the diagnostic value of water T2 alone is limited in terms of discerning muscle abnormalities arising from different neuromuscular diseases such as muscular dystrophies, Charcot-Marie-Tooth neuropathies, or inflammatory myopathies. Clinicians need to be aware of the low specificity that might lead to unnecessary interventions such as muscle biopsy. Second, the VOI delineation is crucial when analyzing water T2, as highlighted by the differences in mean and median water T2, which suggests using the median. Third, confounding pathologies like vascular diseases leading to pooling of fluid in the lower extremities, and effects of exercise prior to the scan must be taken into account when interpreting pathologically elevated T2 values. These effects can be reduced if patients can rest in a lying position for 30 min prior to the MR acquisition [5, 6]. A possible remedy for clinical practice could be performing the MSME acquisition last in the MR protocol such that patients lie still as long as possible. In a previous study of the stability and reproducibility of water T2 measurements in healthy volunteers not restraining physical activity or a resting period before the scan, a slight decrease of water T2 of 1 ms over 1 h was noticed [22]. Since patients in our study were all scanned according to the same MR protocol within 10–20 min after changes of position from standing to supine, the time-dependent changes were systematically low for our whole cohort. Fourth, water T2 relaxometry is a non-standard MR acquisition and requires dedicated post-processing. Several approaches exist [23–25] and are freely available for our protocol [24, 25], but still require off-scanner post-processing and are not certified procedures for diagnostic purposes. It also remains unclear, which method and signal model are best suited. This is especially crucial when selecting the cut-off value, which we set to be at 35 ms based on water T2 values of healthy volunteers measured by Marty et al [19], as we used the same EPG model. The optimal cut-off value, however, is a tradeoff between sensitivity and specificity, which needs further assessment. In general, standardized MR acquisition protocols should be agreed upon to use water T2 relaxometry clinically to facilitate comparison between sites [26].

Our study is of retrospective design representing the clinical routine at our hospital resulting in a rather small and unbalanced cohort. As we considered T1-weighted images as standard for muscle grading, the cohort was further reduced to patients with only early-stage disease for the water T2 analysis. The retrospective setting further generated a selection bias, as only patients with suspected NMDs were included. The low specificity of water T2 might at least partly be attributed to this bias (other than confounding pathologies). For definitive clinical recommendation of water T2 relaxometry, a larger sample size is clearly required. The retrospective setting also does not allow to draw any conclusions on specific NMDs beyond assessing water T2 in different histopathological categories (Fig. 4). These findings are in accordance with general pathophysiologic understandings. Namely, water T2 tends to be higher in inflammatory pattern (suggesting intensive muscle edema, leaky membranes), whereas neurogenic atrophy or myocyte lesions in neurogenic or myopathic diseases do not lead to the same degree of water T2 prolongation. Correlating water T2 with histopathology in human NMDs has only recently been investigated in a relatively small cohort [27], and our study contributes to this topic with data from a rather large cohort. When no alterations in T1-weighted and T2-TIRM images were discernable, we used elevated water T2 to choose the muscle for biopsy. Because of ethical considerations, multiple biopsies could not be performed to rule out this bias. However, that clinical setting is exactly when water T2 relaxometry is advantageous over T2-TIRM images for early detection of NMDs.

In conclusion, we showed an increased sensitivity of quantitative water T2 relaxometry over subjective grading of standard T2-TIRM images for the early detection of NMDs, prior to late-stage fatty infiltration. Therefore, normal-appearing muscle in T2-TIRM images does not rule out disease manifestation in NMDs. Our findings suggest considering quantitative water T2 relaxometry complementary to subjective grading of T2-TIRM images for early detection of NMDs in clinical diagnostic routine.

## Declarations

### Guarantor

The scientific guarantor of this publication is Olivier Scheidegger.

### Statistics and biometry

One of the authors has significant statistical expertise.

### Methodology

• retrospective

• observational

• performed at one institution
